# Dual-probe genome mining identifies citrulassin N, a novel citrulline modified lasso peptide from *Streptomyces* sp. NAX00255

**DOI:** 10.3389/fmicb.2026.1786444

**Published:** 2026-02-24

**Authors:** Zi-Ru Wang, Chao Zeng, Zhang-Yuan Yan, Zi-Fei Xu, Dan Feng

**Affiliations:** 1 Department of Clinical Pharmacy, Affiliated Hospital of Jiangsu University, Zhenjiang, China; 2 State Key Laboratory of Pharmaceutical Biotechnology, Institute of Functional Biomolecules, School of Life Sciences, Nanjing University, Nanjing, China; 3 State Key Laboratory of Phytochemistry and Natural Medicines, Kunming Institute of Botany, Chinese Academy of Sciences, Kunming, China

**Keywords:** biosynthesis, citrulassin, genome mining, lasso peptides, RiPPs

## Abstract

**Introduction:**

Lasso peptides are a structurally distinctive class of ribosomally synthesized and post-translationally modified peptides (RiPPs) featuring a threaded rotaxane topology that confers remarkable thermal stability and protease resistance. Citrulassins represent a rare subgroup of lasso peptides distinguished by a citrulline residue generated through peptidylarginine deiminase (PAD)-catalyzed deimination of arginine. Prior to the identification of citrulassin A, such a modification had not been observed in RiPPs, and notably, the PAD-encoding gene is located outside the canonical lasso peptide biosynthetic gene cluster (BGC).

**Methods:**

Here, we developed a dual-probe genome-mining strategy that integrates homology searches for both the lasso peptide cyclase (CitC) and a PAD homolog to selectively prioritize candidate producers from the IFB bacterial genome database. Guided by this strategy, fermentation and targeted isolation led to the discovery of citrulassin N (1) from *Streptomyces* sp. NAX00255.

**Results:**

Comprehensive structural elucidation using NMR spectroscopy and tandem mass spectrometry confirmed citrulassin N as a novel citrulline-modified lasso peptid.

**Discussion:**

This study expands the structural diversity of citrulline-containing lasso peptides, demonstrates the utility of a dual-probe genome-mining approach for identifying RiPPs with rare post-translational modifications, and provides a practical framework for the targeted discovery of functionally decorated RiPP natural products.

## 1 Introduction

Lasso peptides represent a structurally distinct class of ribosomally synthesized and post-translationally modified peptides (RiPPs), characterized by their unique interlocked threaded-rotaxane topology (Maksimov et al., 2012; Hegemann et al., 2015; Barrett and Mitchell, 2024). This distinctive architecture is formed by an N-terminal macrolactam ring that entraps the C-terminal tail, creating a mechanically interlocked structure (Maksimov and Link, 2014; Cheung-Lee and Link, 2019; Shi et al., 2025). This conformation confers remarkable thermal stability and resistance to proteolytic degradation (Cheung-Lee et al., 2019; Xu et al., 2019; Cheung-Lee et al., 2020). Endowed with such a robust structural scaffold, coupled with their diverse bioactivities including antimicrobial, antiviral, and receptor antagonistic properties, lasso peptides have emerged as promising candidates for natural product discovery and bioengineering research (Wilson et al., 2003; Gavrish et al., 2014; Al Musaimi, 2024; Kolahdoozan and Jahanian-Najafabadi, 2025). Notably, the biosynthetic machinery of lasso peptides is highly streamlined; nonetheless, they encompass an extensive chemical space, driven by the extreme sequence hypervariability within their core peptide regions.

The biosynthesis of lasso peptides is typically encoded by a conserved biosynthetic gene cluster (BGC) comprising a precursor peptide and a minimal set of tailoring enzymes responsible for leader peptide removal and macrolactam ring formation. The canonical pathway involves proteolytic cleavage of the leader peptide, cyclization to form the N-terminal macrolactam ring, and subsequent threading of the C-terminal tail to generate the lasso topology (Maksimov and Link, 2014; Shi et al., 2025). Central to this process is the lasso cyclase, which catalyzes macrolactam formation and enforces the mechanically interlocked architecture (Koos and Link, 2019). In certain cases, additional tailoring enzymes further diversify lasso peptide structures by introducing non-canonical post-translational modifications, thereby expanding their functional and chemical diversity.

**Figure 1:**
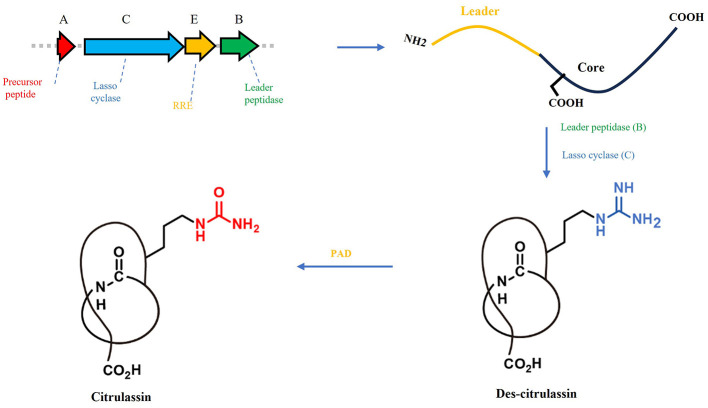
The biosynthetic gene cluster and biosynthetic pathway of citrulassin.

Among the lasso peptide families uncovered through genome mining, the citrulassins constitute a particularly intriguing subgroup. These peptides are distinguished by a rare citrulline residue generated via post-translational deimination of arginine, a reaction catalyzed by peptidylarginine deiminase (PAD) (Tietz et al., 2017; Harris et al., 2020). Citrullination is exceedingly uncommon in bacterial natural products and was unprecedented among RiPPs prior to the discovery of citrulassin A (Tietz et al., 2017). Strikingly, the PAD responsible for this modification is not encoded within the canonical lasso peptide BGC but instead resides elsewhere in the genome (Figure 1), revealing an unusual biosynthetic logic in which a functionally essential tailoring enzyme is genetically disconnected from the core pathway (Harris et al., 2020). This atypical genomic arrangement raises fundamental questions regarding pathway organization, enzyme recruitment, and the hidden chemical potential of RiPP biosynthetic systems.

Traditional activity-guided screening approaches have long been hindered by high rates of compound rediscovery. In contrast, genome mining has emerged as a powerful strategy in the post-genomic era, enabling the systematic identification of biosynthetic gene clusters and the targeted discovery of novel natural products (Winter et al., 2011). The development of dedicated bioinformatic platforms, including antiSMASH, BAGEL, and RODEO, has substantially accelerated the discovery and annotation of RiPP biosynthetic pathways (van Heel et al., 2013; Weber et al., 2015; Tietz et al., 2017). However, identifying RiPPs bearing rare and non-canonical modifications—particularly those catalyzed by enzymes encoded outside their core BGCs—remains challenging using single-probe or motif-based genome-mining strategies.

In this study, we implemented a dual-probe genome-mining strategy to selectively identify citrulassin-type lasso peptides bearing citrulline modifications. Using the lasso cyclase gene (*citC*) from the citrulassin A biosynthetic pathway as an initial probe, we screened the IFB bacterial genome database and identified multiple candidate lasso peptide BGCs (Tietz et al., 2017). Subsequent annotation and classification of the corresponding core peptides were performed using the RiPPMiner platform (Agrawal et al., 2017). To further enrich for citrulline-modified candidates, a confirmed PAD enzyme (WP_064069847.1) was employed as a second probe in a homology-based search (Harris et al., 2020), enabling prioritization of strains harboring both lasso peptide machinery and putative PAD homologs. Guided by this integrated strategy, fermentation and targeted isolation led to the discovery of a previously uncharacterized citrulline-modified lasso peptide, citrulassin N (**1**). Together, this work expands the structural diversity of citrulline-containing lasso peptides and highlights the utility of dual-probe genome mining for the targeted discovery of RiPPs with rare post-translational modifications.

## 2 Materials and methods

### 2.1 General experimental procedures

All chemicals utilized in this study were of analytical grade. Sephadex LH-20 was obtained from GE Biotechnology. Medium-pressure liquid chromatography (MPLC) separations were carried out on a Biotage Isolera One system equipped with a Biotage SNAP Cartridge Ultra C18 column (120 g). Further purification was performed by semipreparative reversed-phase high-performance liquid chromatography (RP-HPLC) using an Agilent 1200 HPLC system fitted with an Agilent Eclipse XDB-C18 column (5 μm, 250 × 9.4 mm). NMR spectra were acquired on a Bruker Avance 400 spectrometer operating at 400 MHz for ^1^H and 100 MHz for ^13^C. High-resolution electrospray ionization mass spectrometry (HRESIMS) data were collected on an Agilent 6530 TOF LC/MS instrument.

### 2.2 Genome mining for novel citrulassin-type lasso peptides

Genome mining was conducted to identify potential strains producing novel citrulassin-type lasso peptides from the IFB database, following a targeted two-step screening strategy integrated with bioinformatic structural prediction. First, the lasso cyclase (*citC*) gene from the citrulassin A BGC was used as the query sequence for BLASTP searches against the genomic sequences of strains in the IFB database. A sequence identity cutoff of >45% was set to retrieve strains harboring homologous *citC*, suggesting the presence of potential lasso peptide BGCs. Second, to enrich for citrulassin-producing candidates, a secondary probe was used: the PAD gene (homologous to WP_064069847.1). This enzyme is distally encoded and critical for citrulline formation during citrulassin A biosynthesis. BLASTP analysis was independently conducted on the genomic data of first-round positive strains to identify those harboring PAD homologs. Strains co-harboring both a lasso peptide BGC with a lasso cyclase C homolog and an extra-cluster PAD homolog were prioritized as potential producers. These dual genetic features are hallmark characteristics of citrulassin biosynthesis, as PAD-mediated arginine deimination is essential for converting arginine to citrulline in mature citrulassins. Finally, the precursor peptides encoded within the candidate BGCs were analyzed using the RiPPMiner tool. This bioinformatic platform was utilized to predict core peptide sequences (following leader peptide cleavage) and tentative structural features of the mature lasso peptides, thereby facilitating the selection of strains with the potential to produce novel citrulassin-type compounds.

### 2.3 Fermentation, extraction, and isolation

The 15 candidate strains were inoculated into 250-ml flasks containing 50 ml TSB medium and cultivated at 30 °C, 220 rpm for 24 h to obtain seed cultures. These seed cultures were then transferred to various screening media and fermented at 30 °C with shaking at 150 rpm. After 7 days of cultivation, XAD-16 resin was added to the fermentation broth to absorb the target products. The resin was washed with deionized water and then extracted three times with methanol. The combined methanol extracts were concentrated under reduced pressure to yield a crude extract. This crude material was initially fractionated by medium-pressure liquid chromatography (MPLC) using a step gradient of CH_3_OH/H_2_O (10–100%, v/v; flow rate 5 ml/min). Collected fractions were analyzed by analytical HPLC and pooled based on their chromatographic profiles, or subjected to further purification via gel column chromatography. Fractions purified by gel column chromatography were re-analyzed. Those containing the target compound were concentrated under reduced pressure, dissolved in methanol, and centrifuged to collect the supernatant. Final separation and purification were achieved by semi-preparative HPLC to obtain pure compounds.

### 2.4 Antimicrobial activity test

To evaluate the inhibitory bioactivity of the lasso peptide citrulassin N (**1**), agar-based inhibition assays were carried out following a previously reported protocol with minor adjustments (Palmer et al., 2018). In brief, bacterial strains were first grown overnight on LB agar plates and then inoculated into LB broth. The cultures were incubated at 37 °C until reaching an OD600 of 0.8. Subsequently, the culture was diluted 1:1,000 into sterilized LB medium containing 0.5% agar that had been pre-warmed to 40 °C, and the mixture was poured into plates. Onto the surface of each solidified soft-agar plate, 10 μl of purified lasso peptide was applied at varying concentrations across a tested gradient. Plates were then incubated at 37 °C for 24 h. Antibacterial activity was determined by measuring the clear inhibition zones formed around the peptide spots. All assays were performed in three independent replicates.

## 3 Results

### 3.1 Genome mining reveals candidate citrulassin-type lasso peptide producers

**Figure 2:**
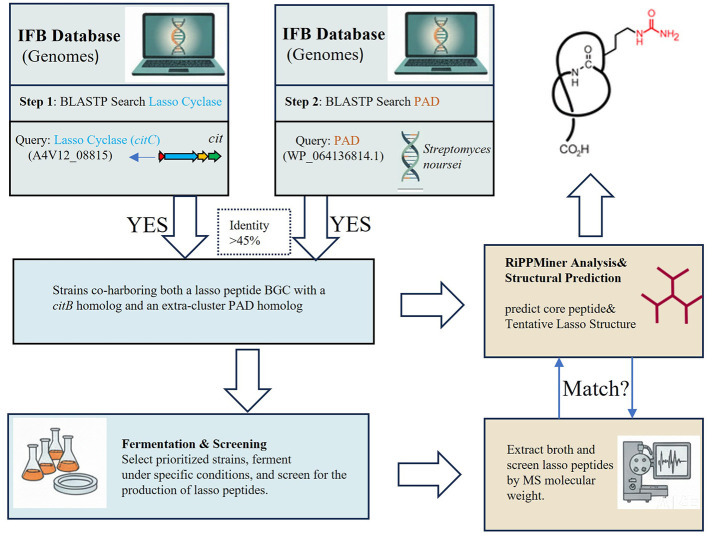
Genome mining for novel citrulassin-type lasso peptide.

A two-step targeted genome mining strategy was employed to identify potential citrulassin-producing strains from the IFB bacterial genome database. In the first round, the lasso peptide cyclase gene *citC* (A4V12_08815) from the citrulassin A biosynthetic gene cluster (BGC) was used as a BLASTP query, applying a sequence identity cutoff of >45%. This search yielded 34 putative lasso peptide BGCs distributed across 34 distinct bacterial strains (Figure 2 and Table 1).

**Table 1:** Characterized citrulassins.

**Bacterial strain**	**Core sequence^α^**	**PAD^β^**
*Streptomyces* sp. NA00298	LLQRSGNDRLILSKN	Y
*Streptomyces decoyicus* CGMCC_4.1861	LLQSSGNDRLILSKN	N
*Streptomyces* sp. NAX00255	LLQSSGNDRLILSKN	Y
*Streptomyces aureocirculatus* CGMCC_4.1609	LLQRHGNDRLILSKN	Y
*Streptomyces* sp. NEAU-S1GS20	LLQRSGNDRLLLSKN	N
*Streptomyces* sp. NA07857	LLGRHGNDRLILSKNS	Y
*Streptomyces aureorectus* DSM 41692	LLGRAGNDRLILSKN	N
*Streptomyces calvus* DSM 40010	LLGRAGNDRLILSKN	N
*Streptomyces alboniger* CGMCC 4.1695	LLARNGNDRLIFSKN	Y
*Streptomyces* sp. NA02069	LLGRHGNDRLILSKN	Y
*Streptomyces* sp. NAK02503	LLQRSGNDRLILSKN	Y
*Streptomyces* sp. NAK5540	LLQRSGNDRLIFSKN	Y
*Streptomyces* sp. NRRL S-337	LLSSSGNDRLILSKN	N
*Streptomyces* sp. NA000687	LLQRSGNDRLVLSKN	N
*Streptomyces lydicamycinicus* NBRC_110027	LLNSSGNDRLVLSKN	N
*Streptomyces* sp. NA01503	LLQRSGRDRLILSKN	Y
*Streptomyces* sp. NA04910	LLQRNGRDRLILSKN	Y
*Streptomyces* sp. NAK02529	LLQRSGNDRLILSKN	N
*Streptomyces* sp. NRRL_S-1448	LLGNSGNDRLLLSKN	N
*Streptomyces* sp. NA04978	LLGHHGNDRLILSKN	Y
*Streptomyces* sp. NA07887	LLGFAGNDRLILSKN	N
*Streptomyces cinerochromogenes* CGMCC_4.1620	LLQRSGNDRLILSKN	N
*Streptomyces durhamensis* CGMCC_4.1699	LLQRHGNDRLIFSKN	Y
*Streptomyces leeuwenhoekii* DSM_42122	LLQRSGNDRLILSKN	N
*Streptomyces* sp. NA07992	LLGKHGNDRLILSKN	N
*Lechevalieria xinjiangensis* CGMCC_4.3525	LLGRSGNDRLILSKN	Y
*Longimycelium tulufanense* CGMCC_4.5737	LLQKNGNDRLILSKN	N
*Amycolatopsis xylanica* KCTC_19581	LLGFSGNDRLILSKN	Y
*Prauserella shujinwangii* CGMCC_4.7125	LLARNGNDRLILSKN	N
*Streptomyces* sp. NAK00080	LLQSSGNDRLILSKN	N
*Streptomyces* sp. NEAU-AAG7	LLGFHGNDRVILSKN	N
*Yuhushiella desert* CGMCC_4.5579	LLQFRGNDRLILSKN	N
*Streptomyces* sp. NAK00032	LLGRHGNDRVVLSKN	Y
*Streptomyces natalensis* NRRL_B-5314	LLEFRGNDRLILSKN	N

^α^Predict core peptide sequences using RiPPMiner; ^β^presence of PAD homologous genes (Y for yes, N for no).

To evaluate whether these BGCs encoded citrulassin-type precursors, the corresponding precursor peptides were analyzed using the RiPPMiner platform, which enables accurate prediction of core peptide sequences and lasso peptide cross-links. Notably, all predicted core peptides shared a conserved arginine residue at position 9, corresponding to the first amino acid immediately outside the macrolactam ring in the lasso topology. This positional conservation is a defining feature of citrulassin-type lasso peptides, as this arginine residue serves as the substrate for post-translational citrullination. In the second round of screening, a confirmed peptidylarginine deiminase (PAD) homolog (WP_064069847.1) was employed as a secondary probe for BLASTP analysis against the 34 first-round positive strains. This analysis identified 15 strains that co-harbored both a citrulassin-like lasso peptide BGC and an extra-cluster PAD homolog (Figure 2 and Table 1). The co-occurrence of a conserved Arg9-containing precursor and a putative PAD enzyme strongly suggests the biosynthetic potential for citrulline-modified lasso peptide production, thereby substantially narrowing the pool of candidate producers.

### 3.2 Fermentation, detection, and isolation of citrulassin N (1)

The 15 candidate strains identified through dual-probe genome mining were subjected to fermentation, followed by metabolite profiling using liquid chromatography–mass spectrometry (LC–MS). Among these strains, *Streptomyces* sp. NAX00255 exhibited a prominent molecular ion peak at *m/z* 820.9511 corresponding to [M+2H]^2+^, which closely matched the molecular weight predicted for a citrulline-modified lasso peptide based on RiPPMiner analysis. Encouraged by this result, large-scale fermentation of *Streptomyces* sp. NAX00255 was carried out to facilitate compound isolation. Subsequent purification using a combination of ODS medium-pressure liquid chromatography (MPLC) and semi-preparative reverse-phase high-performance liquid chromatography (RP-HPLC) afforded 10 mg of the target compound, designated citrulassin N (**1**).

### 3.3 Structural elucidation of citrulassin N (1)

Citrulassin N (**1**) was isolated as a white amorphous powder. High-resolution electrospray ionization mass spectrometry (HR-ESI-MS) established its molecular formula as C_70_H_121_N_21_O_24_, based on the observed [M+2H]^2+^ ion at *m/z* 820.9511 (calcd for C_70_H_123_N_21_O_24_, 820.9519), corresponding to 21 degrees of unsaturation. Although the limited quantity of compound resulted in reduced signal-to-noise ratios for certain NMR spectra, the molecular formula was fully consistent with predictions derived from the associated biosynthetic gene cluster using RiPPMiner. Comprehensive analysis of ^1^H NMR, ^13^C NMR, ^1^H–^1^H COSY, HSQC, HMBC, and MS/MS data enabled unambiguous structural assignment of compound **1**.

The ^1^H NMR (Supplementary Figure S4) and ^1^H–^1^H HSQC (Supplementary Figure S6) spectra showed typical features of a peptide-derived compound, including 16 amide NH signals (δ_H_ 6.80, 6.90, 7.06, 7.16, 7.20, 7.24, 7.28, 7.56, 7.70, 7.80, 7.88, 8.01,8.23, 8.34, 8.42, and 8.92). The ^13^C NMR spectrum (Supplementary Figure S5) also displayed 20 amide/acid carbonyl signals (158.3, 158.4, 159.2, 167.7, 169.4, 169.5, 170.1, 170.8, 171.0, 171.2, 171.6, 172.0, 172.5, 172.8, 173.0, 173.3, 173.6, 173.7, 173.8, and 174.4). Comprehensive analysis of the 1D (^1^H, ^13^C) and 2D (^1^H-^1^H COSY, HSQC, and HMBC) NMR spectra (Supplementary Figures S4–S8) revealed compound **1** contained 15 amino acid residues, including four Leucines (Leu), a Glutamine (Gln), three Serines (Ser), a Glycine (Gly), two Asparagines (Asn), an Aspartic acid (Asp), a Citrulline (Cit), an Isoleucine (Ile), and a Lysine (Lys). Given that 20 carbonyl signals account for 20 of the 21 degrees of unsaturation, we infer the presence of one additional ring structure in the peptide, which aligns with the predicted cyclic architecture of a lasso peptide.

**Figure 3:**
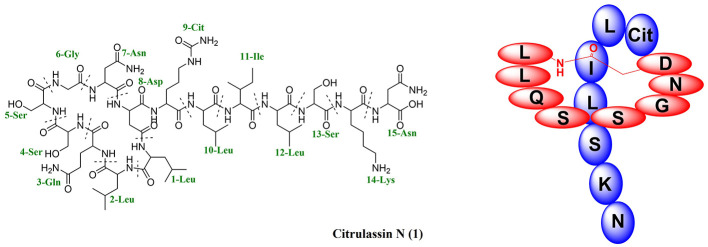
Chemical structures of citrulassin N (**1**).

MS/MS analysis serves as a critical tool for providing direct evidence of amino acid connectivity within peptide chains. LC-MS/MS fragmentation data (Supplementary Figure S1) clearly established the sequence of the C-terminal heptapeptide (Cit9-Leu10-Ile11-Leu12-Ser13-Lys14-Asn15) and corroborated the presence of the macrolactam ring formed by the eight-residue segment (Leu1-Leu2-Gln3-Ser4-Ser5-Gly6-Asn7-Asp8). These results conclusively demonstrate that the planar structure of compound **1** consists of an eight-membered macrolactam ring linked to a seven-residue C-terminal tail, thereby defining its architecture as a lasso peptide (Figure 3).

### 3.4 Proposed biosynthetic pathway of citrulassin N (1)

**Figure 4:**
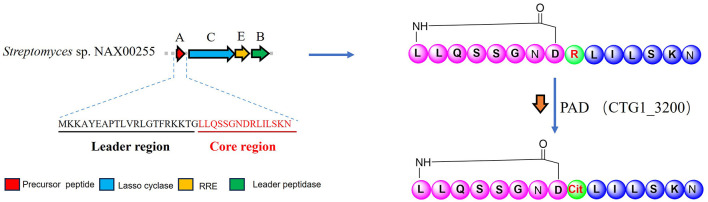
The biosynthetic gene cluster and biosynthetic pathway of citrulassin N (**1**).

Based on genomic analysis of *Streptomyces* sp. NAX00255 and the conserved biosynthetic logic of citrulassins, a plausible biosynthetic pathway of citrulassin N (**1**) was proposed (Figure 4). The precursor peptide encoded within the lasso peptide BGC consists of a leader peptide followed by a 15-residue core peptide (LLQSSGNDRLILSKN), which is consistent with the typical modular architecture characteristic of lasso peptide precursors. The biosynthesis initiates with the proteolytic cleavage of the leader peptide by a leader peptidase, releasing the mature core peptide. Subsequently, the lasso cyclase (a CitC homolog) catalyzes the formation of an eight-membered N-terminal macrolactam ring via amide bond formation between the α-amino group of Leu1 and the side-chain carboxylate of Asp8, a key step in constructing the lasso topology. Notably, the ninth residue in the precursor core peptide is arginine, which is positioned immediately outside the macrolactam ring and serves as the substrate for citrullination. Deimination of Arg9 to citrulline is proposed to be catalyzed by an extra-cluster PAD homolog (WP_064069847.1), yielding the signature citrulline modification that defines citrulassin N. Finally, the C-terminal tail (Leu10-Asn15) threads through the macrolactam ring to form the mechanically interlocked lasso structure, which is consistent with the structural features deduced from NMR and MS/MS data. This biosynthetic pathway closely mirrors that of citrulassin A, underscoring that the conserved combination of a citrulassin-type lasso peptide BGC and an extra-cluster PAD gene is sufficient to direct the biosynthesis of citrulline-modified lasso peptides (Figure 1). These findings further validate the effectiveness of the dual-probe genome-mining strategy for targeted discovery of structurally unique RiPPs.

### 3.5 Antibacterial and cytotoxic activity assays of citrulassin N (1)

Given that several lasso peptides have been reported to exhibit antimicrobial activities (Carson et al., 2023; King et al., 2023; Mucha et al., 2025), the antibacterial potential of citrulassin N (**1**) was evaluated against a panel of Gram-positive (*Staphylococcus aureus* ATCC 6538, *Bacillus subtilis* ATCC 9372) and Gram-negative (*Pseudomonas aeruginosa* ATCC 27853, *Escherichia coli* ATCC 25922) bacterial strains. The results indicated that the compound exhibited only weak inhibitory activity against *Staphylococcus aureus* ATCC 6538, with a minimum inhibitory concentration (MIC) of 40 μg/ml, and showed no significant activity against the other tested strains (Table 2).

**Table 2:** Antimicrobial activities of citrulassin N (**1**).

**Compounds**	***Staphylococcus aureus***	***Bacillus subtilis***	***Pseudomonas aeruginosa***	***Escherichia coli***
	**MIC (**μ**g/ml)**			
Citrulassin N (**1**)	40	>50	>50	>50
Ciprofloxacin^a^	0.195	0.350	2.5	0.195

^a^Positive control.

## 4 Discussion

In this study, we identified and structurally characterized a new citrulline-modified lasso peptide, citrulassin N (**1**), through a targeted genome-mining strategy that integrates two complementary genetic markers. By combining a conserved core biosynthetic enzyme (the lasso peptide cyclase, CitC) with a functionally diagnostic tailoring enzyme (peptidylarginine deiminase, PAD), we were able to efficiently prioritize candidate strains with genuine potential to produce citrulassin-type peptides. This dual-probe strategy substantially reduced the number of candidate biosynthetic gene clusters requiring experimental validation and ultimately enabled the discovery of a previously unreported citrulline-modified lasso peptide.

A defining feature of citrulassins is the presence of a citrulline residue generated through PAD-catalyzed deimination of arginine, a post-translational modification that is exceedingly rare in bacterial natural products. Notably, the PAD responsible for this transformation is encoded outside the canonical lasso peptide biosynthetic gene cluster. The successful identification of citrulassin N reinforces the notion that genetically disconnected tailoring enzymes can play essential roles in RiPP biosynthesis and that their presence in the genome can serve as a reliable indicator of specific chemical modifications. Our results further demonstrate that the conserved Arg residue positioned immediately outside the macrolactam ring represents a privileged site for citrullination, underscoring the tight interplay between lasso peptide topology and post-translational modification.

The excellent agreement between the structure predicted from genome analysis and that experimentally determined by NMR and MS/MS highlights the growing reliability of bioinformatic tools such as RiPPMiner for guiding RiPP discovery. While citrulassin N (**1**) displayed only weak antibacterial activity, its discovery is nonetheless significant. Rather than expanding bioactivity space, this work primarily advances methodological capability by illustrating how genome mining can be tailored to selectively uncover RiPPs bearing rare and chemically distinctive modifications.

More broadly, the dual-probe genome-mining concept demonstrated here is readily extendable to other RiPP families. Pairing core biosynthetic genes with signature tailoring enzymes—particularly those responsible for uncommon or mechanistically intriguing modifications—offers a generalizable strategy for accessing hidden chemical diversity that may be overlooked by conventional single-probe or motif-based searches. As genome databases continue to expand, such focused and logic-driven mining approaches will become increasingly valuable for the efficient discovery of structurally and biosynthetically unusual natural products.

## 5 Conclusion

In conclusion, we report the discovery of citrulassin N (**1**), a new citrulline-modified lasso peptide, through a dual-probe genome-mining strategy that integrates a lasso peptide cyclase and an extra-cluster peptidylarginine deiminase. Comprehensive structural elucidation confirmed both the lasso topology and the site-specific citrulline modification predicted from genomic analysis. This work expands the citrulassin family and demonstrates that targeted genetic filtering based on biosynthetic logic can substantially streamline the discovery of RiPPs with rare post-translational modifications.

## Data Availability

The raw data supporting the conclusions of this article will be made available by the authors, without undue reservation.

## References

[B1] Agrawal P. Khater S. Gupta M. Sain N. Mohanty D. (2017). RiPPMiner: a bioinformatics resource for deciphering chemical structures of RiPPs based on prediction of cleavage and cross-links. Nucleic Acids Res. 45, W80–W88. doi: 10.1093/nar/gkx408 28499008 PMC5570163

[B2] Al Musaimi O. (2024). Lasso peptides realm: insights and applications. Peptides 182:171317. doi: 10.1016/j.peptides.2024.171317 39489300

[B3] Barrett S. E. Mitchell D. A. (2024). Advances in lasso peptide discovery, biosynthesis, and function. Trends Genet. 40, 950–968. doi: 10.1016/j.tig.2024.08.002 39218755 PMC11537843

[B4] Carson D. V. Zhang Y. So L. Cheung-Lee W. L. Cartagena A. J. Darst S. A. . (2023). Discovery, characterization, and bioactivity of the achromonodins: lasso peptides encoded by achromobacter. J. Nat. Prod. 86, 2448–2456. doi: 10.1021/acs.jnatprod.3c00536 37870195 PMC10949989

[B5] Cheung-Lee W. L. Cao L. Link A. J. (2019). Pandonodin: a proteobacterial lasso peptide with an exceptionally long C-terminal tail. ACS Chem. Biol. 14, 2783–2792. doi: 10.1021/acschembio.9b00676 31742991 PMC7010350

[B6] Cheung-Lee W. L. Link A. J. (2019). Genome mining for lasso peptides: past, present, and future. J. Ind. Microbiol. Biotechnol. 46, 1371–1379. doi: 10.1007/s10295-019-02197-z 31165971 PMC6989040

[B7] Cheung-Lee W. L. Parry M. E. Zong C. Cartagena A. J. Darst S. A. Connell N. D. . (2020). Discovery of ubonodin, an antimicrobial lasso peptide active against members of the *Burkholderia cepacia* complex. ChemBioChem 21, 1335–1340. doi: 10.1002/cbic.201900707 31765515 PMC7205569

[B8] Gavrish E. Sit C. S. Cao S. Kandror O. Spoering A. Peoples A. . (2014). Lassomycin, a ribosomally synthesized cyclic peptide, kills mycobacterium tuberculosis by targeting the ATP-dependent protease ClpC1P1P2. Chem. Biol. 21, 509–518. doi: 10.1016/j.chembiol.2014.01.014 24684906 PMC4060151

[B9] Harris L. A. Saint-Vincent P. M. B. Guo X. Hudson G. A. DiCaprio A. J. Zhu L. . (2020). Reactivity-based screening for citrulline-containing natural products reveals a family of bacterial peptidyl arginine deiminases. ACS Chem. Biol. 15, 3167–3175. doi: 10.1021/acschembio.0c00685 33249828 PMC7749083

[B10] Hegemann J. D. Zimmermann M. Xie X. Marahiel M. A. (2015). Lasso peptides: an intriguing class of bacterial natural products. Acc. Chem. Res. 48, 1909–1919. doi: 10.1021/acs.accounts.5b00156 26079760

[B11] King A. M. Zhang Z. Glassey E. Siuti P. Clardy J. Voigt C. A. (2023). Systematic mining of the human microbiome identifies antimicrobial peptides with diverse activity spectra. Nat. Microbiol. 8, 2420–2434. doi: 10.1038/s41564-023-01524-6 37973865

[B12] Kolahdoozan M. Jahanian-Najafabadi A. (2025). Lasso peptides: a focus on therapeutic index. World. J. Microbiol. Biotechnol. 41:151. doi: 10.1007/s11274-025-04374-y 40289244

[B13] Koos J. D. Link A. J. (2019). Heterologous and in vitro reconstitution of fuscanodin, a lasso peptide from Thermobifida fusca. J. Am. Chem. Soc. 141, 928–935. doi: 10.1021/jacs.8b10724 30532970 PMC6475475

[B14] Maksimov M. O. Link A. J. (2014). Prospecting genomes for lasso peptides. J. Ind. Microbiol. Biotechnol. 41, 333–344. doi: 10.1007/s10295-013-1357-4 24142336

[B15] Maksimov M. O. Pan S. J. James Link A. (2012). Lasso peptides: structure, function, biosynthesis, and engineering. Nat. Prod. Rep. 29, 996–1006. doi: 10.1039/c2np20070h 22833149

[B16] Mucha P. Ruczynski J. Prochera K. Rekowski P. (2025). Lasso peptides—a new weapon against superbugs. Int. J. Mol. Sci. 26:8184. doi: 10.3390/ijms26178184 40943111 PMC12427821

[B17] Palmer J. D. Piattelli E. McCormick B. A. Silby M. W. Brigham C. J. Bucci V. (2018). Engineered probiotic for the inhibition of salmonella via tetrathionate-induced production of microcin H47. ACS Infect. Dis. 4, 39–45. doi: 10.1021/acsinfecdis.7b00114 28918634 PMC5766358

[B18] Shi J. Zhang Y. Ren W. Q. Shi Y. Wei Y. Y. Zhang B. . (2025). Biosynthesis of collinodin unveils iterative oxidative and prenylation modifications. ACS Catal. 15, 6628–6639. doi: 10.1021/acscatal.5c01005

[B19] Tietz J. I. Schwalen C. J. Patel P. S. Maxson T. Blair P. M. Tai H. C. . (2017). A new genome-mining tool redefines the lasso peptide biosynthetic landscape. Nat. Chem. Biol. 13, 470–478. doi: 10.1038/nchembio.2319 28244986 PMC5391289

[B20] van Heel A. J. de Jong A. Montalban-Lopez M. Kok J. Kuipers O. P. (2013). BAGEL3: automated identification of genes encoding bacteriocins and (non-)bactericidal posttranslationally modified peptides. Nucleic Acids Res. 41, W448–W453. doi: 10.1093/nar/gkt391 23677608 PMC3692055

[B21] Weber T. Blin K. Duddela S. Krug D. Kim H. U. Bruccoleri R. . (2015). antiSMASH 3.0-a comprehensive resource for the genome mining of biosynthetic gene clusters. Nucleic Acids Res. 43, W237–W243. doi: 10.1093/nar/gkv437 25948579 PMC4489286

[B22] Wilson K. A. Kalkum M. Ottesen J. Yuzenkova J. Chait B. T. Landick R. . (2003). Structure of microcin J25, a peptide inhibitor of bacterial RNA polymerase, is a lassoed tail. J. Am. Chem. Soc. 125, 12475–12483. doi: 10.1021/ja036756q 14531691

[B23] Winter J. M. Behnken S. Hertweck C. (2011). Genomics-inspired discovery of natural products. Curr. Opin. Chem. Biol. 15, 22–31. doi: 10.1016/j.cbpa.2010.10.020 21111667

[B24] Xu F. Wu Y. Zhang C. Davis K. M. Moon K. Bushin L. B. . (2019). A genetics-free method for high-throughput discovery of cryptic microbial metabolites. Nat. Chem. Biol. 15, 161–168. doi: 10.1038/s41589-018-0193-2 30617293 PMC6339573

