# Supplementary material for: Dual-probe genome mining identifies citrulassin N, a novel citrulline modified lasso peptide from Streptomyces sp. NAX00255
Source: Front Microbiol. 2026 Feb 24;17:1786444. doi: 10.3389/fmicb.2026.1786444 (PMC12971885; doi:10.3389/fmicb.2026.1786444)
Supplement: Supplementary file 1 [file Supplementary_file_1.docx]

***Supplementary Material***

**Dual-Probe Genome Mining Identifies** **Citrulassin N, a Novel Citrulline Modified Lasso Peptide from *Streptomyces sp*. NAX00255**

Zi Ru Wang^1,2^, Chao Zeng^1^, Zhang Yuan Yan^2^, Dan Feng^1*^, Zi Fei Xu^2,3*^

^1^ Department of Clinical Pharmacy, Affiliated Hospital of Jiangsu University, Zhenjiang, 212001, China.

^2^ State Key Laboratory of Pharmaceutical Biotechnology, Institute of Functional Biomolecules, School of Life Sciences, Nanjing University, Nanjing 210023, China.

^3^ State Key Laboratory of Phytochemistry and Natural Medicines, Kunming Institute of Botany, Chinese Academy of Sciences, Kunming 650201, China.

*** Correspondence**

Dan Feng, [dandanfung@163.com](mailto:dandanfung@163.com)

Zi Fei Xu, [xuzifei@mail.kib.ac.cn](mailto:xuzifei@mail.kib.ac.cn)

**List of Supporting Information**

**Supplementary tables:**

**Table S1.** Deduction of gene functions in the *cit* cluster from *Streptomyces* sp. NAX00255

**Supplementary figures:**

**Figure S1.** HR-MS/MS data of **1** indicating the order of amino acids connections.

**Figure S2.** HR-ESI-MS(+ESI) spectrum of Citrulassin N (**1**).

**Figure S3.** HR-ESI-MS(-ESI) spectrum of Citrulassin N (**1**).

**Figure S4.** ^1^H NMR spectrum (600 MHz) of Citrulassin N (**1**) in DMSO-*d*_6_.

**Figure S5** ^13^C NMR spectrum (150 MHz) of Citrulassin N (**1**) in DMSO-*d*_6_.

**Figure S6.** HSQC spectrum of Citrulassin N (**1**) in DMSO-*d*_6_.

**Figure S7.** HMBC spectrum of Citrulassin N (**1**) in DMSO-*d*_6_.

**Figure S8.** ^1^H-^1^H COSY spectrum of Citrulassin N (**1**) in DMSO-*d*_6_.

**Table S1.** Deduction of gene functions in the *cit* cluster from *Streptomyces* sp. NAX00255


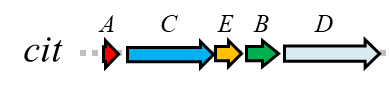


| **ORF** | **Amino acids** | **Blastp homologue** | **Identity/ coverage [%]** | **Protein** |
| --- | --- | --- | --- | --- |
| *citA* | 36 | Precursor peptide | 97/100 | GAA2524653.1 |
| *citC* | 612 | Putative asparagine synthetase | 28/67 | WTZ13469.1 |
| *citE* | 86 | lasso peptide biosynthesis PqqD family chaperone | 74.4/98 | HLL35107.1 |
| *citB* | 144 | lasso peptide biosynthesis B2 protein | 80/99 | MCF6523395.1 |
| *citD* | 705 | ABC transporter ATP-binding protein | 91/97 | WP_079158224.1 |


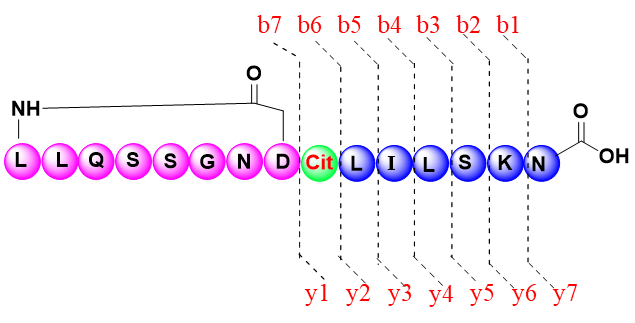


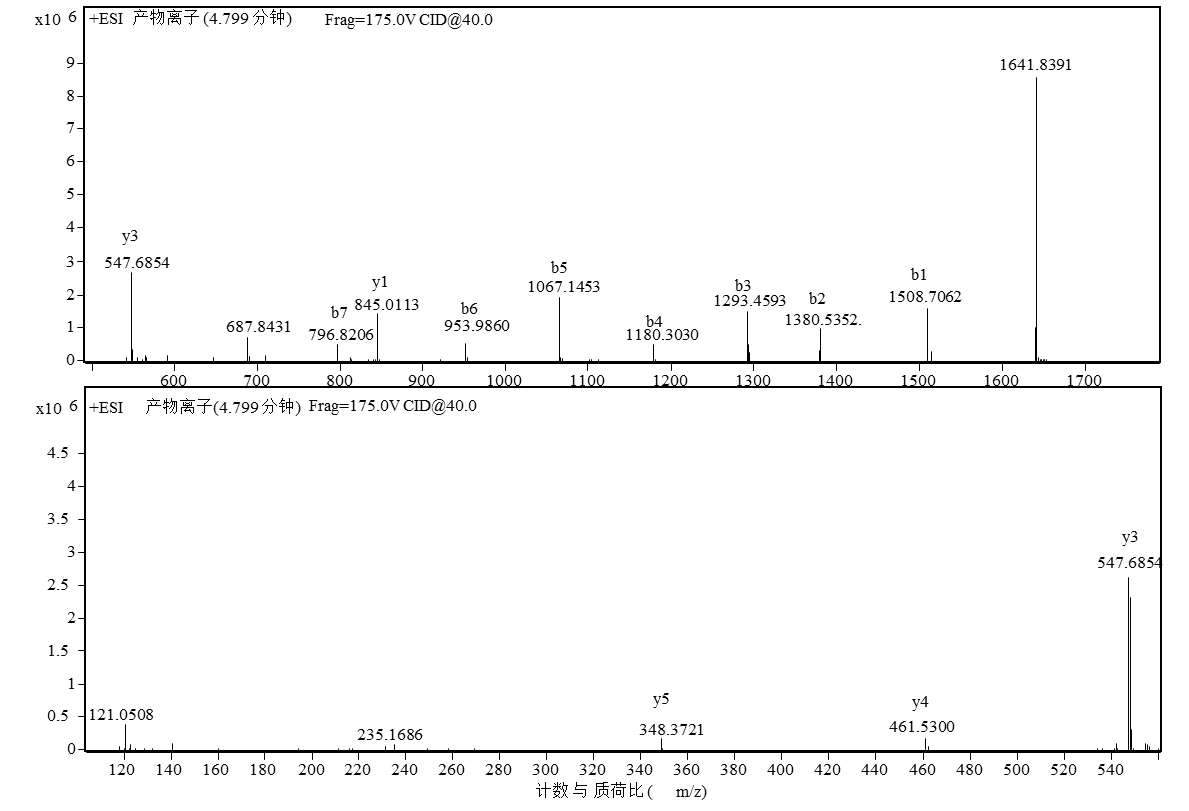


| Species | Calculated Mass/Da | Observed Mass/Da | Species | Calculated Mass/Da | Observed Mass/Da |
| --- | --- | --- | --- | --- | --- |
| b1 | 1508.7068 | 1508.7062 | y1 | 845.0118 | 845.0113 |
| b2 | 1380.5357 | 1380.5352 | y2 | 687.8425 | 687.8431 |
| b3 | 1293.4589 | 1293.4593 | y3 | 574.6859 | 574.6854 |
| b4 | 1180.3024 | 1180.3030 | y4 | 461.5294 | 461.5300 |
| b5 | 1067.1459 | 1067.1453 | y5 | 348.3728 | 348.3721 |
| b6 | 953.9853 | 953.9860 | y6 | 261.2960 | Not obs.. |
| b7 | 796.8210 | 796.8206 | y7 | 133.1250 | Not obs. |

**Supplementary** **Figure S1.** HR-MS/MS data of **1** indicating the order of amino acids connections.


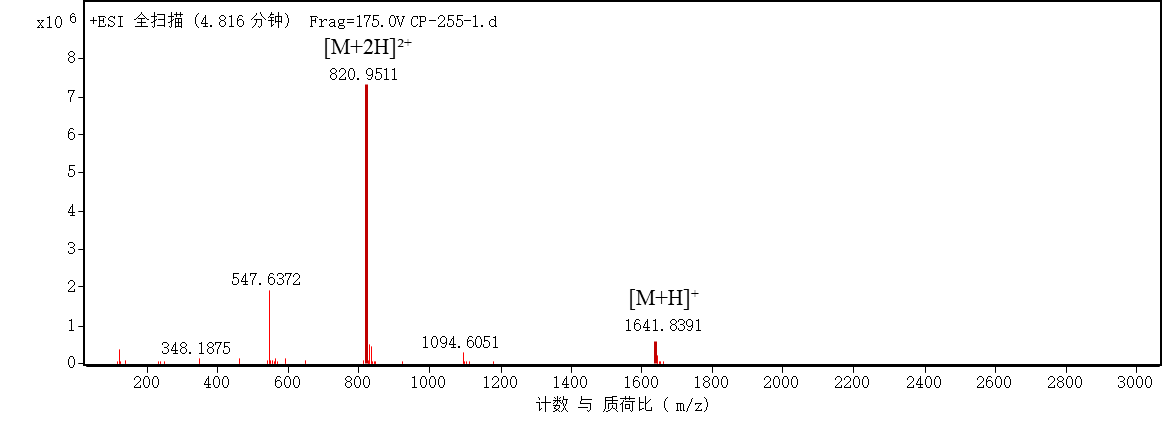


**Supplementary** **Figure S2.** HR-ESI-MS(+ESI) spectrum of Citrulassin N (**1**).


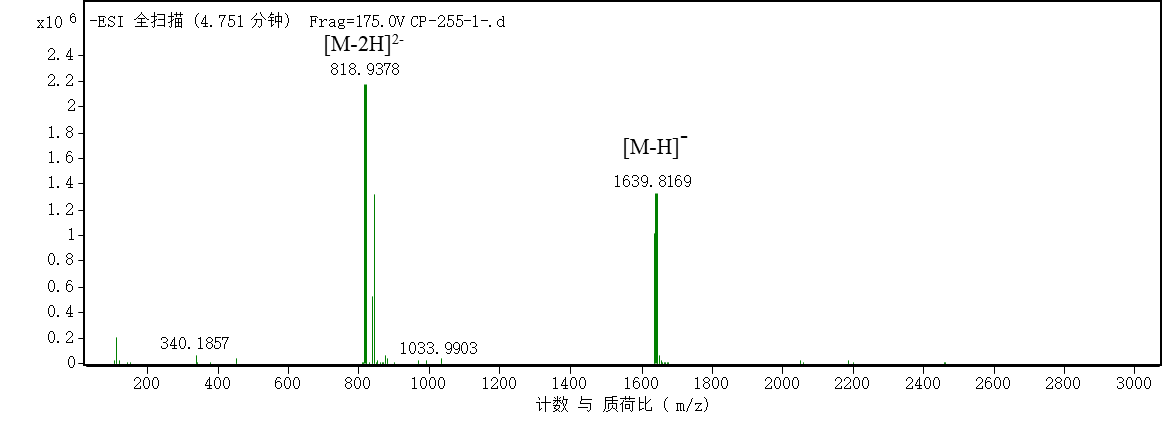


**Supplementary Figure S3.** HR-ESI-MS(-ESI) spectrum of Citrulassin N (**1**).

**
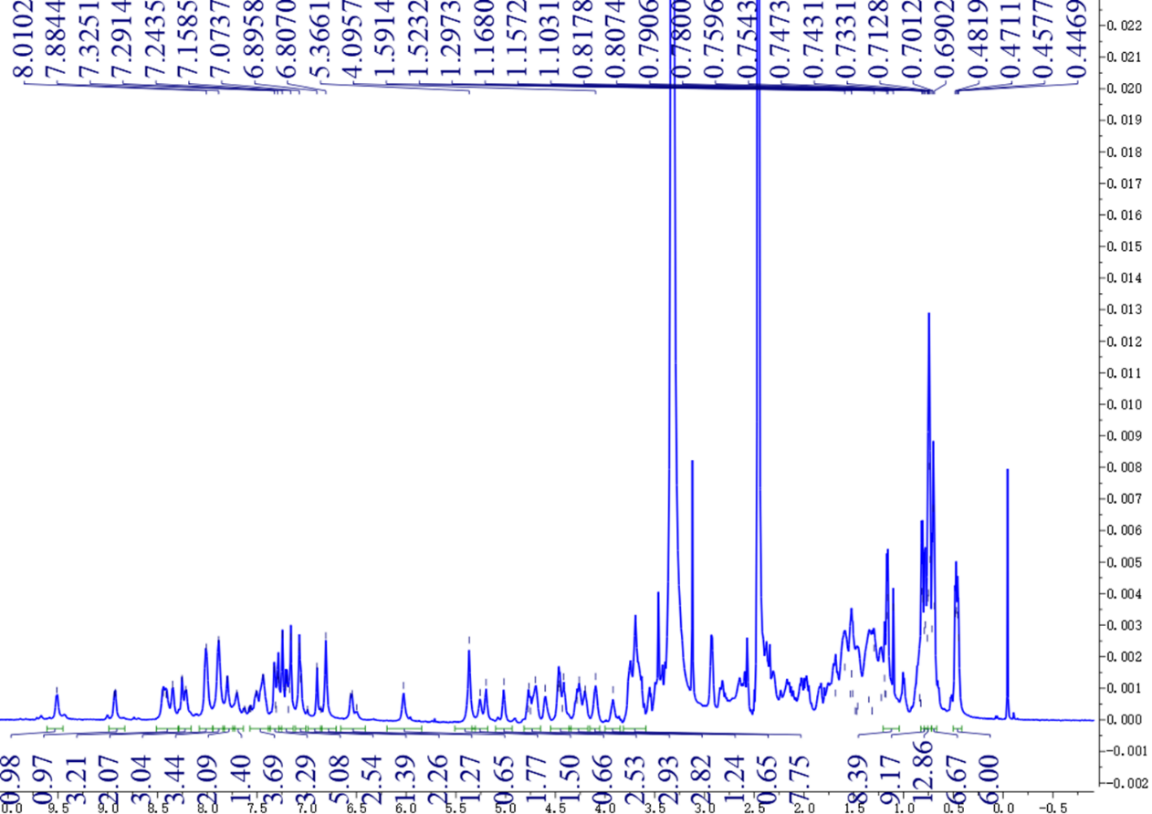
**

**Supplementary Figure S4.** ^1^H NMR spectrum (600 MHz) of Citrulassin N (**1**) in DMSO-*d*_6_.


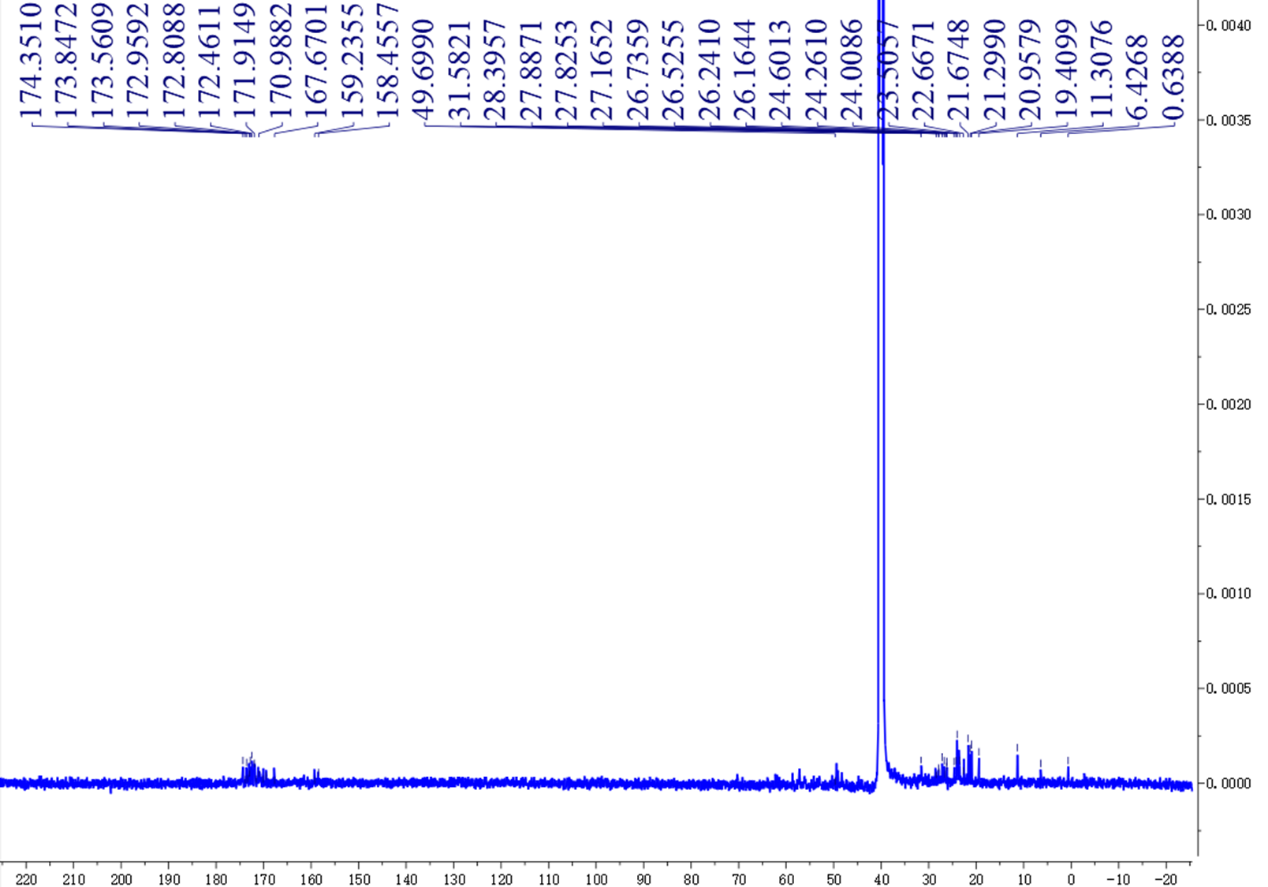


**Supplementary Figure S5** ^13^C NMR spectrum (150 MHz) of Citrulassin N (**1**) in DMSO-*d*_6_.


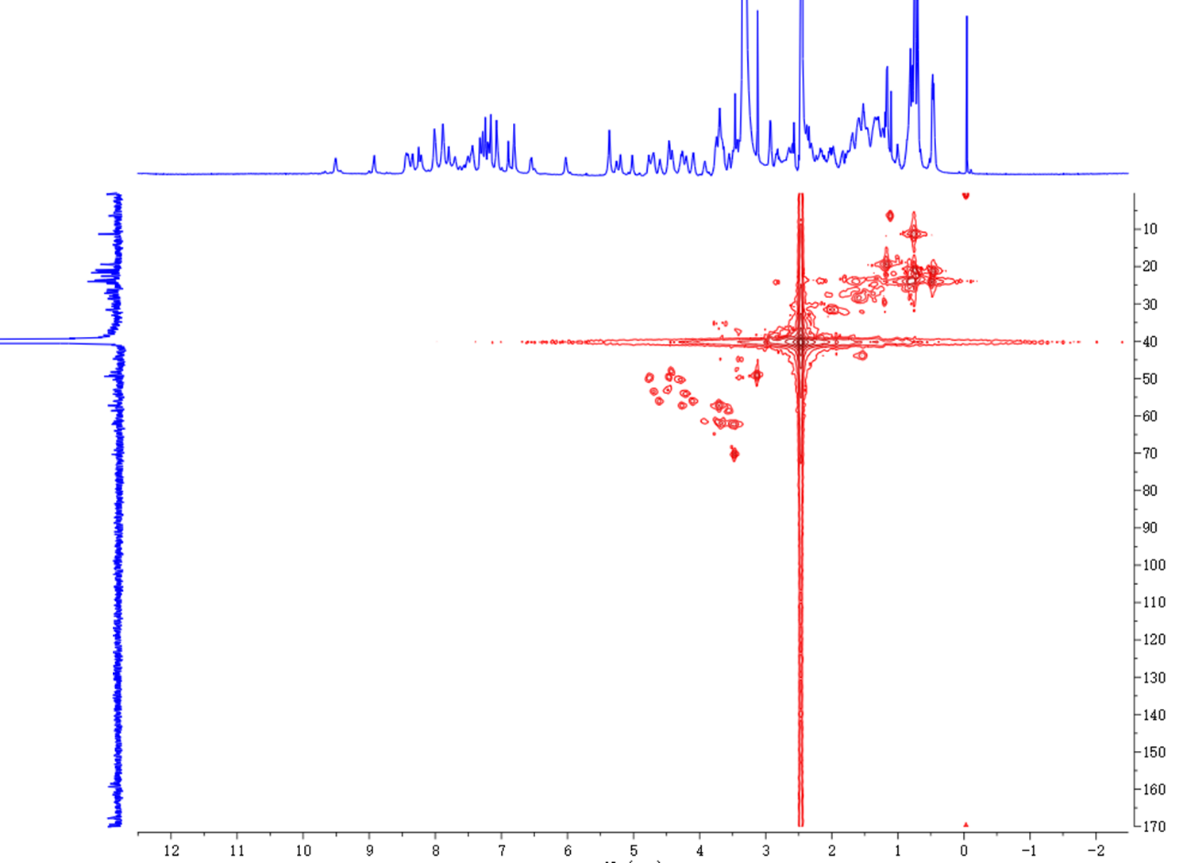


**Supplementary** **Figure S6.** HSQC spectrum of Citrulassin N (**1**) in DMSO-*d*_6_.

**
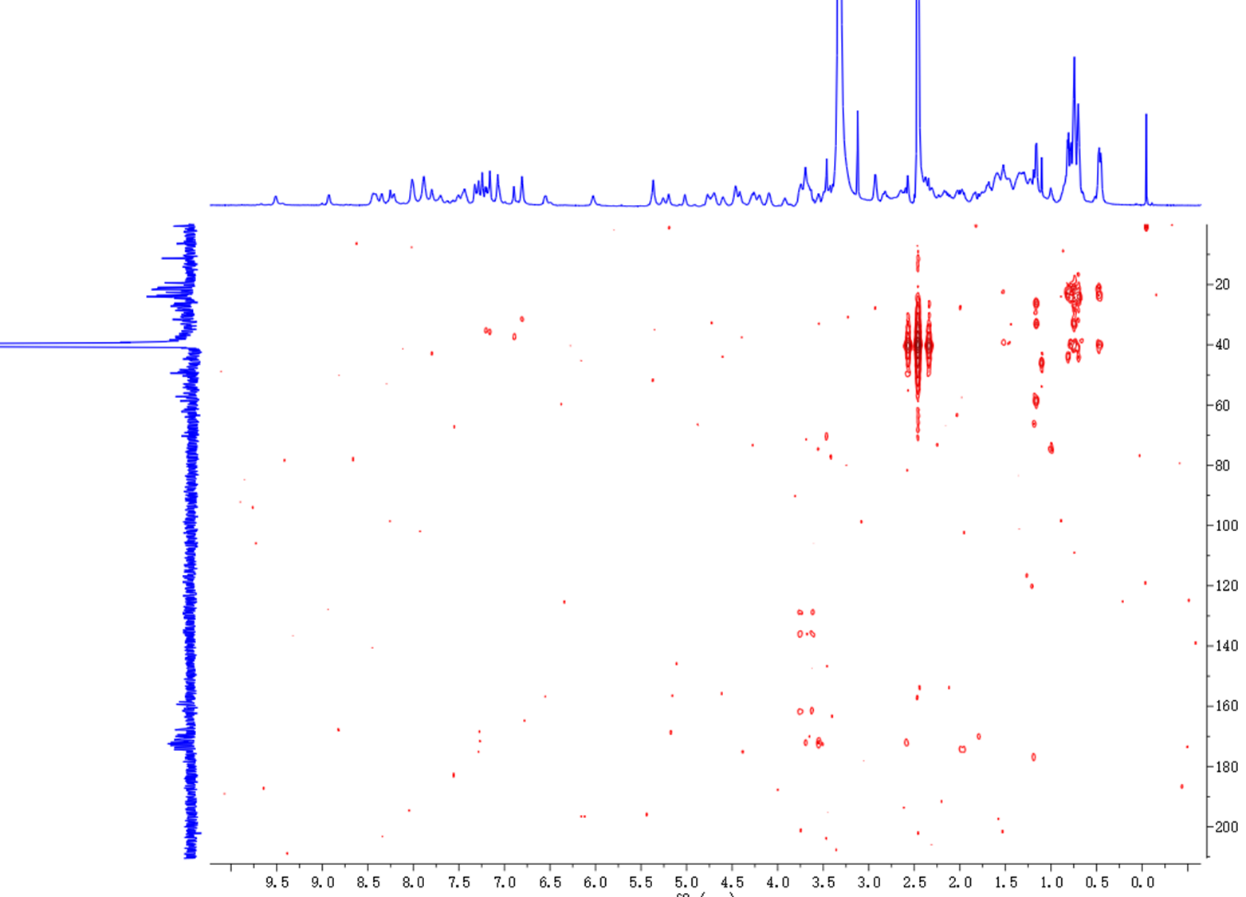
**

**Supplementary Figure S7.** HMBC spectrum of Citrulassin N (**1**) in DMSO-*d*_6_.


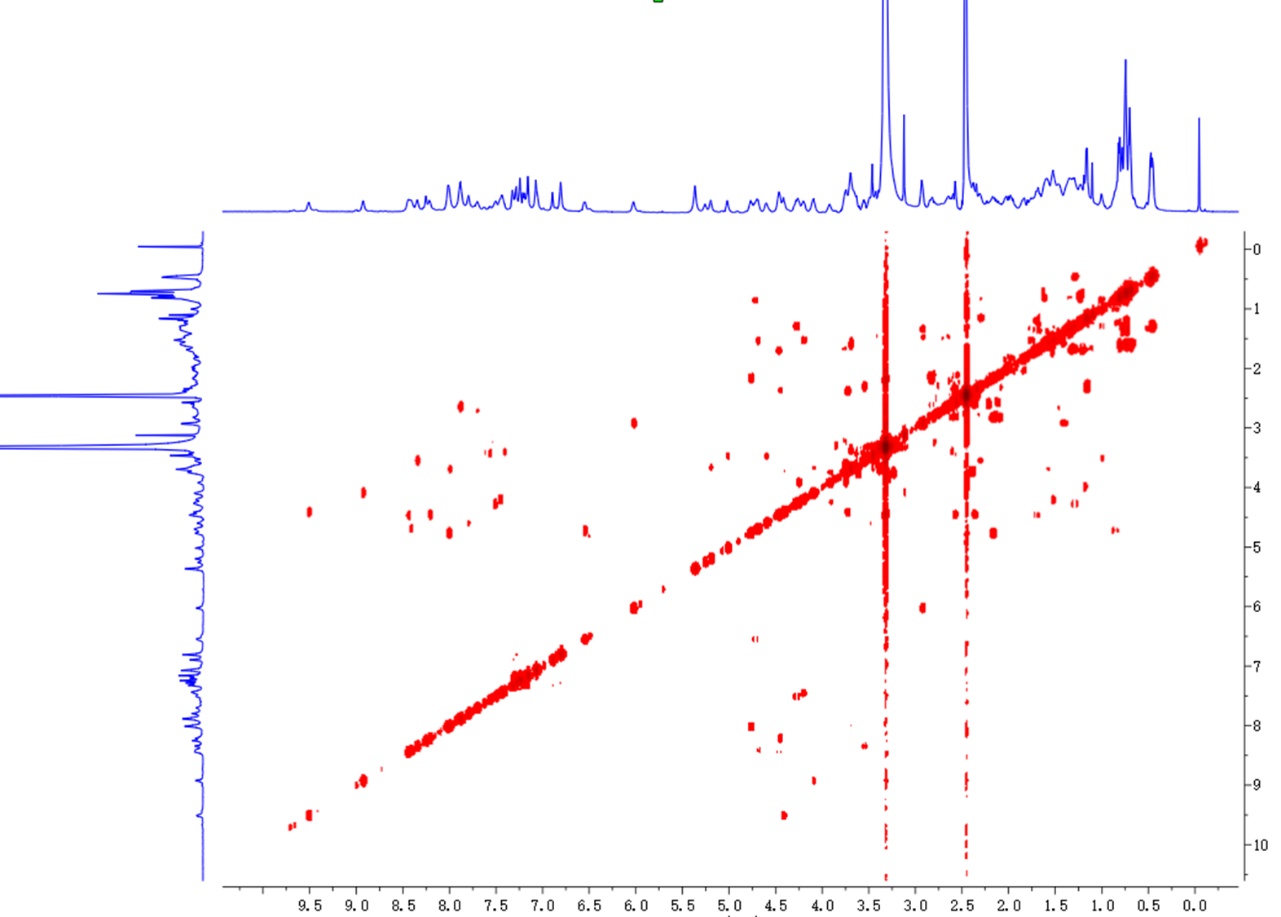


**Supplementary Figure S8.** ^1^H-^1^H COSY spectrum of Citrulassin N (**1**) in DMSO-*d*_6_.
